# An amyloidogenic hexapeptide derived from amylin attenuates inflammation and acute lung injury in murine sepsis

**DOI:** 10.1371/journal.pone.0199206

**Published:** 2018-07-10

**Authors:** Sidharth Mahapatra, Lihua Ying, Peggy Pui-Kay Ho, Michael Kurnellas, Jonathan Rothbard, Lawrence Steinman, David N. Cornfield

**Affiliations:** 1 Department of Pediatrics, University of Nebraska Medical Center, Omaha, Nebraska, United States of America; 2 Department of Pediatrics, Stanford University School of Medicine, Stanford, California, United States of America; 3 Department of Neurology and Neurological Sciences, Stanford University School of Medicine, Stanford, California, United States of America; 4 Alector, South San Francisco, California, United States of America; National Institutes of Health, UNITED STATES

## Abstract

Although the accumulation of amyloidogenic proteins in neuroinflammatory conditions is generally considered pathologic, in a murine model of multiple sclerosis, amyloid-forming fibrils, comprised of hexapeptides, are anti-inflammatory. Whether these molecules modulate systemic inflammatory conditions remains unknown. We hypothesized that an amylin hexapeptide that forms fibrils can attenuate the systemic inflammatory response in a murine model of sepsis. To test this hypothesis, mice were pre-treated with either vehicle or amylin hexapeptide (20 μg) at 12 hours and 6 hours prior to intraperitoneal (i.p.) lipopolysaccharide (LPS, 20 mg/kg) administration. Illness severity and survival were monitored every 6 hours for 3 days. Levels of pro- (IL-6, TNF-α, IFN-γ) and anti-inflammatory (IL-10) cytokines were measured via ELISA at 1, 3, 6, 12, and 24 hours after LPS (i.p.). As a metric of lung injury, pulmonary artery endothelial cell (PAEC) barrier function was tested 24 hours after LPS administration by comparing lung wet-to-dry ratios, Evan’s blue dye (EBD) extravasation, lung histology and caspase-3 activity. Compared to controls, pretreatment with amylin hexapeptide significantly reduced mortality (p<0.05 at 72 h), illness severity (p<0.05), and pro-inflammatory cytokine levels, while IL-10 levels were elevated (p<0.05). Amylin pretreatment attenuated LPS-induced lung injury, as demonstrated by decreased lung water and caspase-3 activity (p<0.05, versus PBS). Hence, in a murine model of systemic inflammation, pretreatment with amylin hexapeptide reduced mortality, disease severity, and preserved lung barrier function. Amylin hexapeptide may represent a novel therapeutic tool to mitigate sepsis severity and lung injury.

## Introduction

In neurodegenerative conditions like Alzheimer’s disease (AD), accumulation of extracellular β-amyloid (Aβ) is a hallmark of disease [1]. In Aβ plaques, microglia, astrocytes, complement proteins, and cytokines, like TNF-α, TGF-β, and IL-1β, can be found [2–5]. Similarly, in multiple sclerosis (MS), the accumulation of amyloid-forming proteins, such as serum amyloid protein (SAP), amyloid P protein (APP), aβ crystallin (HspB5), and tau, are found in demyelinating plaques [6, 7]. The evolution of these amyloidogenic plaques activates a T-cell mediated myelin-specific autoimmune response, signaling lymphocytes and macrophages to infiltrate the central nervous system (CNS) and directly damage axons [8, 9].

However, a study designed to explore the putative pro-inflammatory function of Aβ revealed anti-inflammatory properties. Specifically, exogenous Aβ administration led to a reduction of Th_1_ and Th_17_-mediated autoimmunity in a mouse model of multiple sclerosis, experimental autoimmune encephalomyelitis (EAE), as motor paralysis, lymphocyte activation, and CNS inflammation were decreased [1]. Similarly, HspB5 had therapeutic benefit in animal models of multiple sclerosis, stroke, and cardiac and retinal ischemia-reperfusion injury [10–14]. Consistent with the notion that these molecules can confer protection from inflammation and CNS injury, in mice wherein HspB5 and Aβ were genetically deleted, paralysis and inflammation associated with EAE were increased [1, 12]. Similar potentiation of disease was reported in mice modified to delete genes encoding other amyloid-forming proteins, such as PrP, SAP, and tau [15–17].

Eisenberg and others have established that the dry steric zipper, which is the basis for the 3-D crossed β strand characteristic of amyloid fibrils, can be formed by peptides as short as six amino acids [18–20]. With hexapeptides, the zipper interface is formed via the association of side chains from two extended β pleated sheets, a structure that affords simplicity, homogeneity, solubility, and less cytotoxicity than the intact parent proteins [21]. Previous work in our lab with a set of amyloidogenic hexapeptides derived from tau, Aβ, PrP, HspB5, amylin, SAP, and insulin B chain showed that these hexameric fibrils lowered IL-6 levels and ameliorated clinical paralysis in mice afflicted with EAE with no apparent toxicity [6].

Molecular characterization of HspB5 has refined the collective understanding of the anti-inflammatory properties of amyloidogenic hexapeptides. A region corresponding to residues 73–92 was shown to form fibrils, possess chaperone activity and reduce paralysis and neuroinflammation [22]. Moreover, residues falling outside this area neither formed fibrils nor possessed chaperone activity, thus confirming the critical importance of the amyloid fibril structure in modulating neuroinflammation [23]. By mass spectroscopy, over 70 ligands were identified to precipitate with HspB5 and Tau_623-628_, half of which were acute phase proteins or members of the complement and coagulation cascades, including C3, clusterin, and transthyretin [6, 24].

More recent studies have revealed another mechanism for the attenuation of neuroinflammation by amyloidogenic hexapeptides. In EAE, intraperitoneal injections of amyloid fibrils (tau_623-628_ or amylin_28-33_) led to the activation and exodus of B-1a lymphocytes (CD19^hi^CD5^+^CD23^-^) from the peritoneum to draining lymph nodes where IL-10, an immune-suppressive cytokine, was produced. In fact, IL-10 knock-out or B-cell deficient mice with EAE failed to respond to amyloid fibrils; adoptive transfer of B-1a cells in these mice, in turn, restored therapeutic efficacy [25]. Prior studies have established the anti-inflammatory role of these IL-10-producing B-cells in inflammatory bowel disease, lupus, and allergic airway disease [26–29]. In each of these autoimmune diseases, reduction in symptoms correlated with lower peak levels of TNF-α, IFN-γ, and IL-6, a pattern reminiscent of the immune suppressive effects of amyloidogenic hexapeptides.

Whether the mechanisms that underlie the therapeutic efficacy of amyloidogenic hexapeptides in neuroinflammatory conditions might be operative in systemic inflammation remains unknown. To address the potential that amyloidogenic hexapeptides possess significant anti-inflammatory properties in the context of systemic inflammation with evidence of end-organ damage, we studied the effect of a specific amyloid-forming hexapeptide, amylin_28-33_, in a murine model of sepsis. Our findings suggest that amyloidogenic hexapeptides have the capacity to mitigate systemic inflammation.

## Materials and methods

### Animals

Adult 8 to 10-week-old female wild type (WT) C57BL/6J (B6) mice purchased from Jackson Laboratories (Sacramento, CA) were housed in the Research Animal Facility at Stanford University. Animal experiments were approved by and performed in compliance with the National Institute of Health guidelines of the Institutional Animal Care and Use Committee (IACUC) at Stanford University.

### Reagents

Lipopolysaccharide (LPS from Escherichia coli O111:B4, batch number 014M4019V) and Evan’s blue dye (EBD) were obtained from Sigma-Aldrich (St. Louis, MO). Freshly synthesized amylin hexapeptide reconstituted in PBS at a concentration of 100 ug/mL was synthesized at Stanford. OPTEIA ELISA kits for mouse TNF-α, IFN-γ, IL-6, and IL-10 were purchased from BD Pharmigen (San Jose, CA). Antibodies were obtained from Jackson ImmunoResearch (West Grove, PA).

### Peptide synthesis

Peptides were synthesized using solid-phase techniques and commercially available Fmoc amino acids, resins, and reagents (PE Biosystems and Bache) on an Applied Biosystems 433A peptide synthesizer, as previously described [30]. Purity of the peptides was shown to be greater than 90% using a PE Biosystems 700E HPLC and a reverse-phase column (Alltech Altima). The molecular weight of the peptides was confirmed using matrix-assisted laser desorption mass spectrometry.

### LPS endotoxemia model

#### Lipopolysaccharide dose determination

Mice were inoculated with LPS (i.p.) at doses of 5 mg/kg, 10 mg/kg, 20 mg/kg and 40 mg/kg; control mice received PBS. Mice were assessed ever 6 hours for 60 hours. Percent survival was determined for each group.

#### Sepsis induction

Mice were pre-treated with 20 μg amylin (i.p.) at 12 h and 6 h prior to induction of endotoxemia; control mice received no amylin pretreatment. All mice were subsequently inoculated with LPS (i.p.) at a dose of 20 mg/kg (time 0). Illness severity and mortality were assessed and recorded at regular intervals over a 3-day period. Mice alive at the end of the experiment were euthanized.

#### Illness severity scoring

Scores were assigned using a recently validated illness severity scoring system [31]. Mice were assessed every 6 hours for the first 24 hours, then every 12 hours for the next 24 hours, then every 24 hours for the next 24 hours. Briefly, scores were given for the following domains of murine health: coat appearance, level of consciousness, activity level, response to stimulus, eye appearance, respiratory rate, and respiratory quality. Mice were scored on a scale of 0–4, with elevated scores being given for progressively poorer health. Mice were euthanized for scores ≥21.

#### Enzyme-linked immunoassays

Cytokine Elisa assays (BD Pharmigen, San Jose, CA) for TNF-α, IFN-γ, IL-6, and IL-10 were performed on plasma collected via retro-orbital blood collection [32] at 1 h, 3 h, 6 h, 12 h, and 24 h after LPS (i.p.). Mice were euthanized after plasma collection. Elisa plates were blocked with 3% BSA in PBS with 0.05% Tween 20 (Sigma, St. Louis, MO) overnight at 4°C. Subsequent incubations and washes were performed at room temperature. The next day, plates were washed and incubated for 2 h with murine serum samples diluted between 1:200 and 1:800 depending on the cytokine assay, from either mice with LPS-induced endotoxemia or healthy controls. Plates were washed and bound antibodies (Abs) in sera were detected using HRP-conjugated secondary reagents specific for murine IgG (H chain) Abs diluted to 1:10,000 (Jackson ImmunoResearch, West Grove, PA). The colorimetric reaction was developed with 3,39,5,59-tetramethylbenzidine (1-step Ultra TMB; ThermoScientific), stopped with 1 M sulfuric acid, and quantitated using a SpectraMax spectrometer (Molecular Devices). Given the large number of samples per time point (10 mice per time point x 5 time points = 50 mouse per experiment), blood collections were done on separate days for control and amylin pretreatment groups.

### Acute lung injury (ALI) model

#### Lung injury induction

Mice were pre-treated with 20 μg amylin (i.p.) for 6 hours, followed with either vehicle (PBS) or 20 mg/kg LPS (i.p.) (time 0). Animals were allowed to recover overnight before further injection with EBD via retroorbital injections at doses of 30 mg/kg, at 30 min before termination of the 24 h LPS treatment. At the end of exposure, lungs were harvested after intravascular perfusion with PBS.

#### Evan’s blue dye measurement

EBD was extracted in 100 mg lung weight/ml formamide (Sigma) at 70°C water bath overnight. EBD concentrations were measured as previously described [33]. In brief, EBD standards were prepared at 1, 5, 10, 20 and 25 mg/ml in formamide solution. Standards and samples loaded in 96-well plates (200 μl/well) were read at 620 nm/740 nm wavelength. Corrected OD_620_ was calculated using the Garcia correction formula:

$${\text{Corrected}\;\text{A}}_{620}={\text{Observed}\;\text{A}}_{620}–\left(\left(\text{slope}*{\text{Observed}\;\text{A}}_{740}\right)+\text{intercept}\right)$$

Concentration was calculated using y = a + bx (where a = intercept, b = slope).

#### Lung fixation

Lungs were fixed at 25 cm H_2_O pressure with 10% formalin and paraffin embedded for histology. Frozen sections of adult mice were obtained by first inflating the lungs with a 1:1 ratio of PBS and optimal cutting temperature compound (OCT)-embedding medium (Sakura Finetek, Torrance, CA) at a pressure of 35 cm H_2_O, then immersed in OCT, and snap frozen before cryosectioning. Images were acquired using a BZ-9000 (Keyence, Itasca, IL, USA) microscope using Brightfield 20x objective and a BZ-II Analyzer (Keyence). For the H&E staining we used the standard H&E process (Thermo Scientific).

#### Wet-to-dry lung weight ratio

The lung wet-to-dry (W/D) ratio was used as an index of lung water accumulation after lung injury. To measure wet lung weight, lungs were dissected while mice were under deep sevoflurane anesthesia, with weight measured immediately after excision. Lung tissue was then dried in an oven at 70°C for up to 5 days or until tissues were completely dry, and weighed again to determine the dry weight. The W/D weight ratio was calculated by dividing the wet by the dry weight as described previously [34].

#### Caspase-3 activity

For caspase 3 staining, we used cleaved Caspase-3 antibody from Cell Signaling at 1:500 dilution (Cat # ASP175, Danvers, MA) with overnight incubation at 4°, then detected with anti-rabbit-HRP and DAB. For evaluating regions with positive caspase 3 staining, ImageJ, a processing and analysis software, was used, as described previously [35]. In brief, digital images were acquired with a microscope equipped with a 3.0-megapixel digital camera (Olympus; Melville, NY). The regions of interest were photographed in 8-bit grayscale at a magnification of 20x, the backgrounds were normalized, and the density thresholds were set to 130 (minimum) and 255 (maximum). The image then was inverted to give the positive staining as red on a black background. Analysis was performed using the ImageJ particle analysis algorithm to detect caspase-3 positive staining. The percent positive area was calculated by dividing the positive caspase-3 staining area by the total area of the image.

#### Statistical analysis

Mortality data are presented in Kaplan-Meier curve format as aggregate survival, and statistical significance was assessed by log-rank test to detect differences between groups (n = 18). Illness severity data are presented as aggregate means ± SEM of separate experiments, and statistical significance was assessed by a Mann-Whitney U-test to detect differences between groups (n = 13–15). Cytokine data and caspase-3 activity are presented as mean ± SD, and statistical significance was assessed by two-tailed, unpaired Student’s T-test to detect differences between groups (n = 10); of note, due to high mortality by 24 hours, the sample sizes decreased throughout the study period. Lung barrier function data are presented as mean ± SEM, and statistical significance was assessed by one-way ANOVA and Bonferroni’s multiple comparisons test to detect differences between groups (n = 10). A p-value of 0.05 or lower was considered significant.

## Results

### Optimal dose for LPS-induced endotoxemia was 20 mg/kg in our mouse model for sepsis

**Fig 1:**
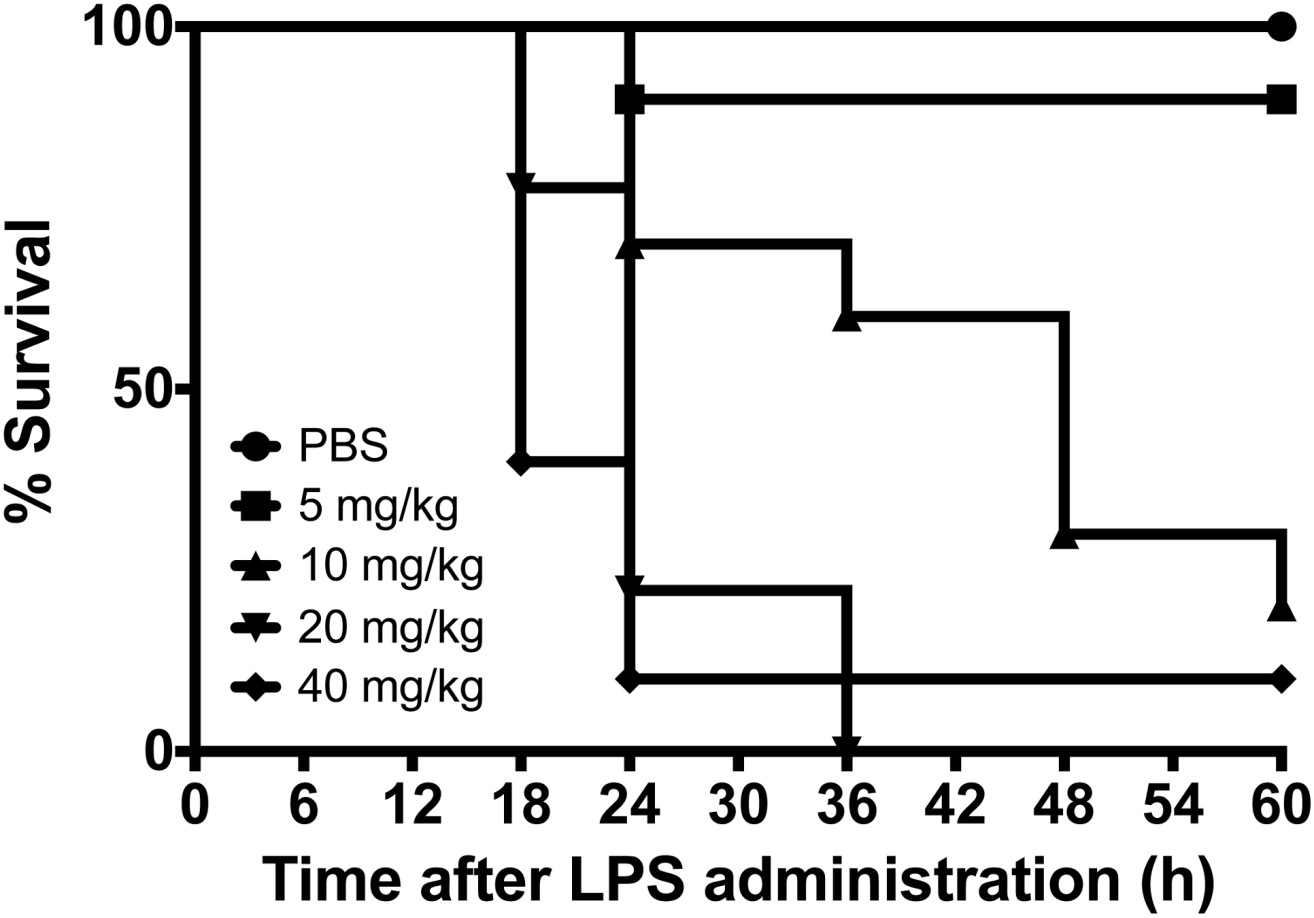
Determination of optimal LPS dose for the induction of systemic inflammation in mice. Adult female B6 mice were inoculated with LPS (i.p.) at doses ranging from 5 mg/kg to 40 mg/kg at time 0; control mice received vehicle (PBS). Mice were assessed every 6 hours for up to 60 hours. Percent survival was determined for each group and presented in Kaplan-Meier format. Each group started with 10 mice.

To be able to show a discriminatory effect of amylin hexapeptide on survival, we first sought to determine an optimal dose of LPS (i.p.) that would predictably lead to high mortality in mice. Reports of lethal doses of LPS range from 10 mg/kg to 40 mg/kg [36–38]. We therefore selected doses of LPS ranging from 5 mg/kg up to 40 mg/kg. Expectedly, a dose of 5 mg/kg did not differ markedly in survival from control with a mortality rate at 60 hours of 10%. However, doses from 10 mg/kg to 40 mg/kg showed stepwise mortality starting between 18–24 hours for the duration of the experiment (Fig 1). From these data, we chose an LPS dose of 20 mg/kg for all subsequent experiments to induce high illness severity and mortality in mice.

### Pretreatment with amylin_28-33_ attenuated illness severity and decreased overall mortality

Kurnellas *et al*. demonstrated that administration of 10 μg of amyloidogenic fibril (i.p.) in their model of EAE triggered an exodus of B-1a lymphocytes from the peritoneum to secondary lymphoid organs where the primary anti-inflammatory cytokine, IL-10, was produced; this process took approximately 4 hours [25]. We chose to investigate the effects of a higher dose of amylin (20 μg) at 2 pretreatment time points (6 hours and 12 hours prior to LPS inoculation).

**Fig 2:**
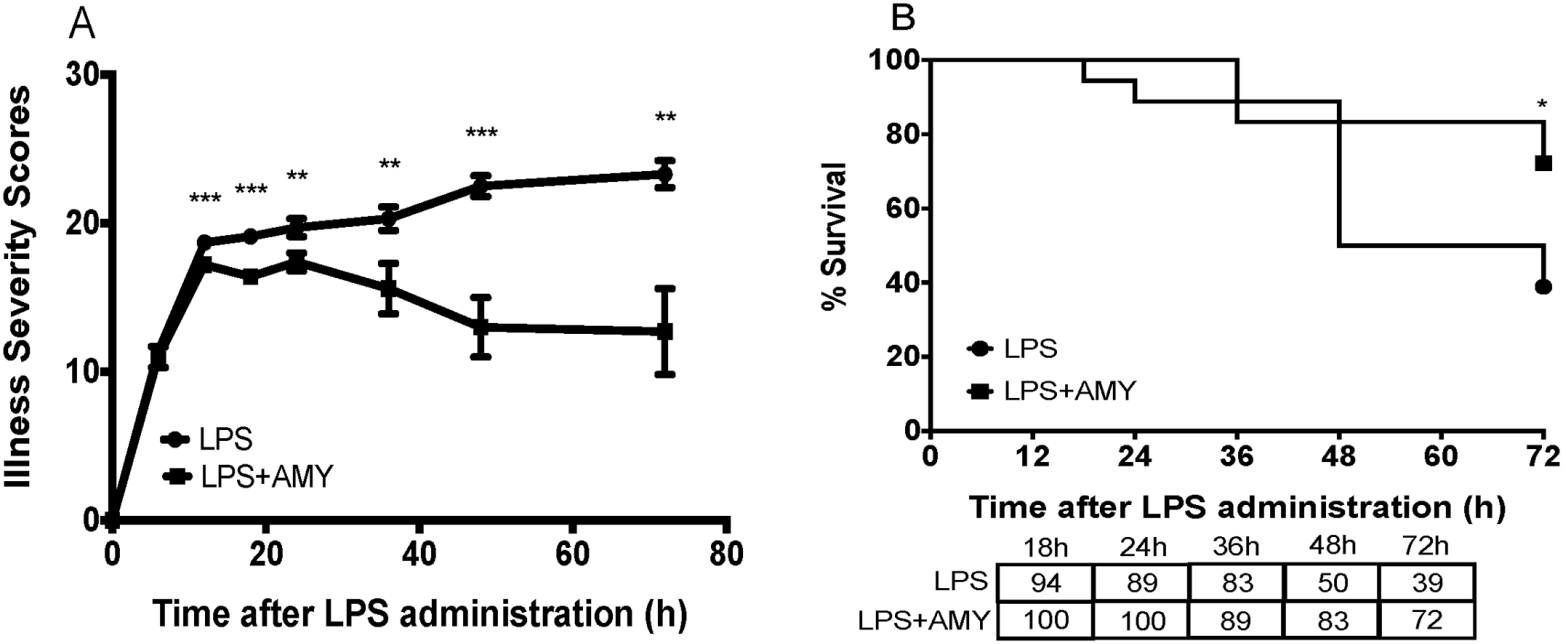
Pretreatment with amylin reduces illness severity and mortality in an LPS-induced systemic inflammatory mouse model. Adult female B6 mice were pretreated with 20 μg amylin at 12 h and 6 h prior to i.p. administration of LPS; control mice received no pretreatment. All mice were subsequently inoculated with LPS (i.p.) at a dose of 20 mg/kg at time 0. Illness severity (A) and mortality (B), which were regularly assessed over a 3-day period, were significantly reduced in the pretreatment group. Aggregate data are presented as mean ± SEM, n = 13–15 mice per data point, * p<0.05 ** p<0.01 *** p<0.001 by Student’s t-test between groups.

In control animals, illness severity worsened throughout the course of the experiment, whereas in the pretreatment group, peak illness severity occurred 12 h after LPS (i.p.) administration (17.4 ± 0.6) and improved thereafter. (Fig 2A). Illness severity scores were lower in the pretreatment group (p<0.05, versus control) at all time points except at 6 hours after LPS (i.p.) administration. Finally, in contrast with the control group wherein illness severity worsened throughout the duration of the experiment, illness severity decreased in the pretreatment group with an average 72 h score of 12.7 ± 2.9 versus 23.3 ± 0.9 in the control group. Amylin pretreatment also reduced overall mortality. At 72 hours after LPS (i.p.) administration, the mortality rate was 61% in the control group compared to 28% in the pretreatment group (Fig 2B, p<0.05). Taken together, illness severity and mortality were lower and the duration of peak illness was shorter in amylin pre-treated animals (S1 Video).

### Pretreatment with amylin_28-33_ decreased pro-inflammatory cytokine expression and increased the expression of the anti-inflammatory cytokine, IL-10

**Fig 3:**
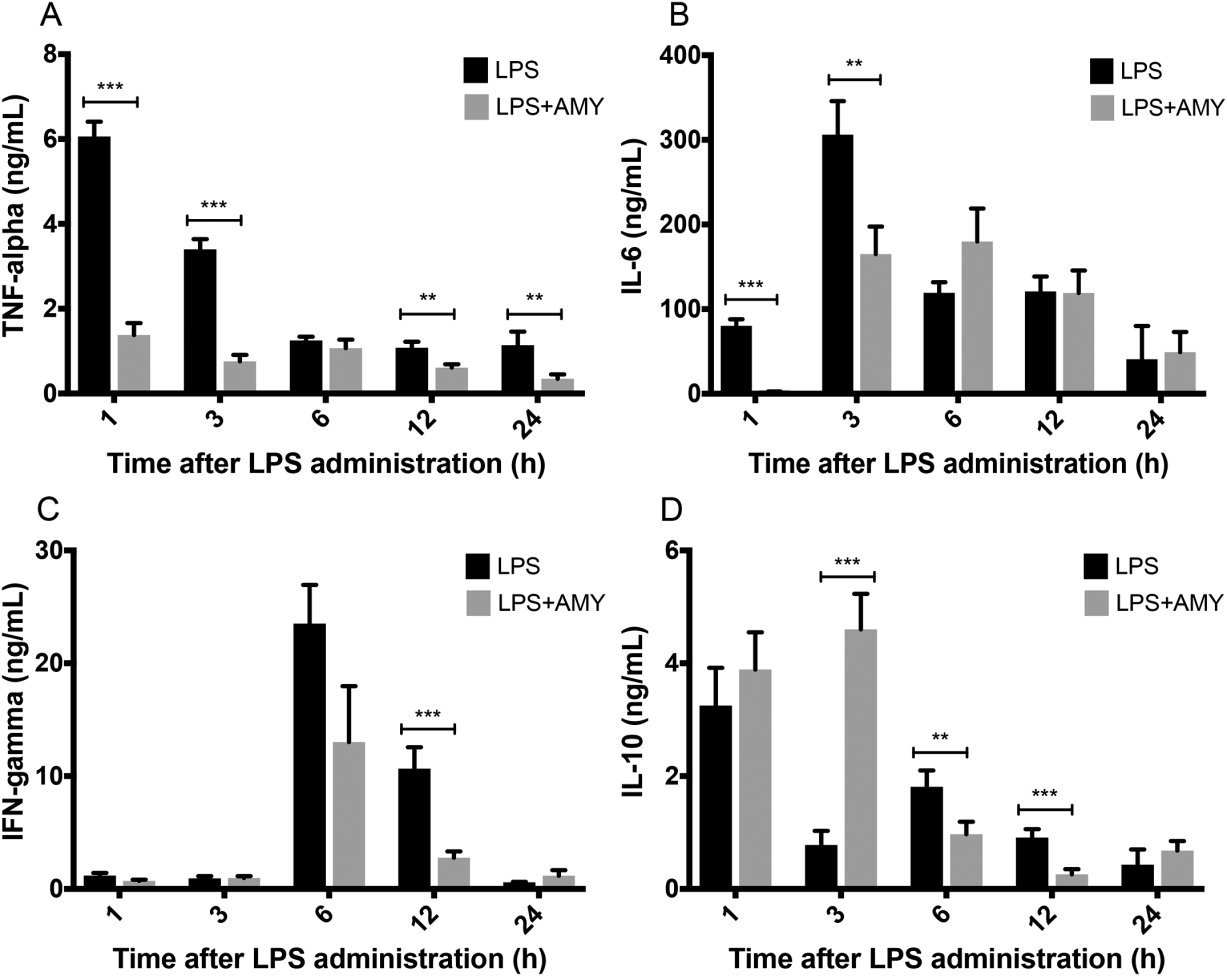
Pretreatment with amylin reduces TNF-α, INF-γ, and IL-6 while sustaining IL-10 levels in an LPS-induced systemic inflammatory mouse model. Adult female B6 mice, with or without amylin pretreatment, underwent induction of systemic inflammation as described prior. Cytokine Elisa assays for TNF-α (A), IL-6 (B), IFN-γ (C), and IL-10 (D) were performed on plasma collected at 1 h, 3 h, 6 h, 12 h, and 24 h after LPS (i.p.) administration. Pretreatment with amylin reduced pro-inflammatory cytokines (TNF-α, IFN-γ, and IL-6), while sustaining IL-10 levels at 3 h after LPS-endotoxemia induction. Aggregate data are presented as mean ± SEM, n = 10 mice per data bar, * p<0.05 ** p<0.01 *** p<0.001 by Student’s t-test between groups.

To further elucidate the mechanism of immune suppression, cytokine profiles were studied at regular intervals after LPS (i.p.) administration. Levels of the pro-inflammatory cytokines, TNF-α, IFN-γ, and IL-6, were higher in control, compared to pretreatment group. TNF-α was the first cytokine to rise and peaked at 1 hour. In amylin hexapeptide pretreated animals, TNF-α expression was 4-fold reduced at 1 and 3 hours after LPS (i.p.) administration (Fig 3A, p<0.001), and TNF-α remained lower for the duration of the experiment (Fig 3A, p<0.01 at 12 and 24 hours after LPS (i.p.) administration). In controls, IL-6 began to rise at 1 hour and peaked at 3 hours; however, in pre-treated animals, IL-6 levels did not begin to rise until 3 hours and peaked at 6 hours after LPS (i.p.) administration. Moreover, in pre-treated animals the level at 3 hours was less than half of controls. (Fig 3B, p<0.01). IFN-γ peaked at 6 hours in both groups. A 4-fold reduction in the pretreatment group was noted at 12 hours after LPS (i.p.) administration (Fig 3C, p<0.001). Notably, IL-10 levels peaked 1 hour after LPS (i.p.) administration in both groups. However, the elevation was sustained in the pretreatment group for 3 hours after LPS (i.p.) administration and was almost 6-fold higher than in LPS exposed animals without amylin pretreatment (Fig 3D, p<0.001). Thereafter, in the pretreatment group, IL-10 levels dropped below control.

### Pretreatment with amylin_28-33_ preserved lung barrier function and decreased caspase-3 activity in the lungs

**Fig 4:**
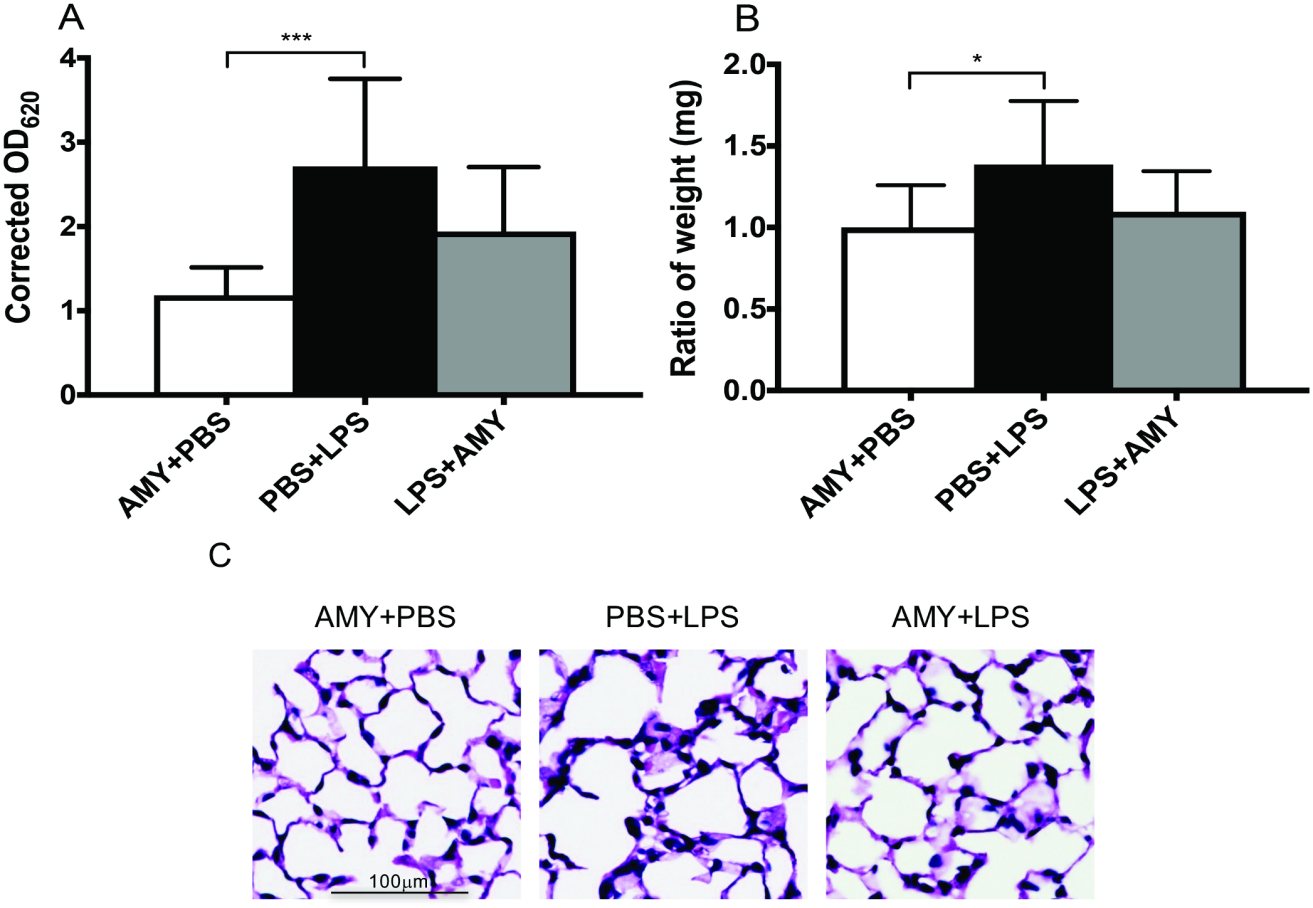
Pretreatment with amylin attenuates lung injury in an LPS-induced systemic inflammatory mouse model. Adult female B6 mice, with or without amylin pretreatment, underwent induction of systemic inflammation as described prior. At 24 hours after LPS (i.p.) administration, lung tissues were inflated, fixed and evaluated for lung injury. (A) Evans Blue Dye (EBD) incorporation in lung tissue was determined by retro-orbital injection of EBD 30 min prior to the 24h experimental endpoint in PBS-only, LPS, and LPS+amylin treated mice. (B) Excessive lung water accumulation was measured using the wet/dry weight ratios of lung tissues. All values presented are mean ± SEM, with n = 10 mice/group. *p<0.05 **p<0.005 ***p<0.001 vs. PBS, via 1-way ANOVA and Bonferroni’s multiple comparisons test. Both EBD and lung water accumulation were significantly elevated in the LPS group and did not differ between PBS and LPS+AMY groups. (C) Representative H and E staining of lungs at 20X magnification from mice treated with PBS+AMY, LPS+PBS, or LPS+AMY. In animals treated with LPS, air space disease was evident with fluid in the alveoli, increased interstitial prominence, and hyaline membrane formation. In animals that received amylin pretreatment, air space disease and lung water was decreased, compared to LPS. n = 5 mice/group.

**Fig 5:**
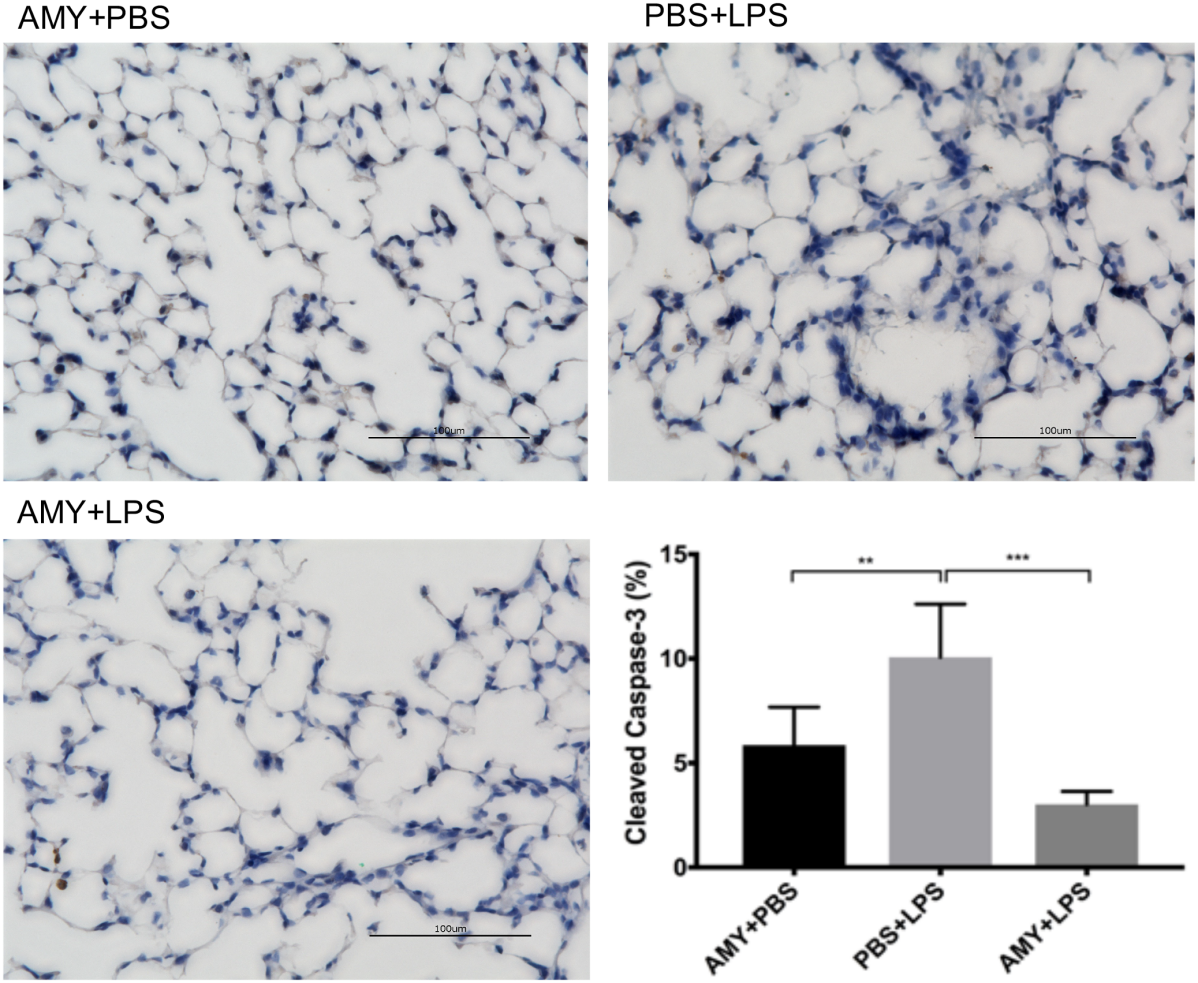
Pretreatment with amylin reduces lung cell apoptosis in an LPS-induced systemic inflammatory mouse model. Adult female B6 mice, with or without amylin pretreatment, underwent induction of systemic inflammation as described prior. At 24 hours after LPS (i.p.) administration, lung tissues were inflated, fixed and evaluated for apoptosis using cleaved caspase-3 activity as a metric. Representative images were taken at 40X magnification. Animals pretreated with LPS demonstrated increased cleaved caspase-3 activity. This increase was blocked by pretreatment with amylin. Aggregate data presented as mean ± SEM, n = 5 mice per data bar, *p<0.05 **p<0.005 ***p<0.001 vs. adult PBS, via 1-way ANOVA and Bonferroni’s multiple comparisons test.

Lung injury was evaluated 24 hours after LPS administration in control and pretreatment groups by examining dye extravasation, lung weight wet-to-dry ratios, lung histology, and caspase-3 activity. In LPS-only treated animals, EBD extravasation and the wet-to-dry ratio were both increased 24 hours after LPS treatment (p<0.05, versus baseline). In contrast, in the pretreatment group, neither EBD extravasation nor wet-to-dry ratios differed from baseline values (Fig 4A and 4B). Moreover, qualitative evaluation of lung histology demonstrated more fluid-filled alveoli and increased interstitial prominence in LPS-treated, as compared to either PBS- or LPS+amylin pretreated mice (Fig 4C). In addition, caspase-3 activity was significantly lower in the pretreatment group compared to the LPS only group and did not differ from baseline values (Fig 5).

## Discussion

The data in this report support the proposition that the hexapeptide amylin_28-33_ possesses physiologically significant systemic anti-inflammatory properties. In a murine model of intraperitoneal endotoxemia, pretreatment with amylin_28-33_ decreased illness severity, mortality, and systemic inflammation. More specifically, in the pretreatment group, a significantly lower peak illness severity score was reached by 12 hours, with a durable and favorable effect on illness severity throughout the study period. Moreover, the risk of mortality was demonstrably decreased in animals that were pretreated with the hexapeptide amylin_28-33_ TNF-α has been cited to be amongst the most important mediators of mortality associated with LPS [39]. Data demonstrating that the pro-inflammatory cytokines, TNF-α, IFN-γ, and IL-6, were decreased while expression of the anti-inflammatory cytokine, IL-10, was sustained over the first several hours after LPS exposure in animals that received the hexapeptide amylin_28-33_ offer mechanistic insight into the observed benefits in mortality and clinical severity. Further evidence of physiologic benefit derives from data demonstrating that lung injury is attenuated in animals pretreated with the hexapeptide amylin_28-33_ Taken together, these findings provide evidence that the hexapeptide amylin_28-33_ can modify the systemic inflammatory response even in the presence of a powerful pro-inflammatory insult.

These findings are significant as this represents the first evidence that these peptides can modify a general immune response, not simply autoimmune and neuroinflammatory conditions. The present findings mirror those of Kurnellas *et al*. in EAE wherein amyloidogenic fibrils decreased pro-inflammatory cytokines, i.e. TNF-α, IFN-γ, and IL-6, specifically by triggering the migration of IL-10-producing B-1a lymphocytes to secondary lymphoid organs [25]. While B-1a lymphocytes were not isolated in our study, the differential expression of IL-10 in pre-treated mice supports this mechanism of inflammation mitigation. The present studies in a model of LPS-induced systemic inflammation provide strong support for the notion that these fibrils possess physiologically relevant anti-inflammatory properties and, when considered in concert with prior literature [25, 27] and IL-10 expression, that B-1a lymphocytes may facilitate this effect. The present results raise the potential that these peptides might be therapeutically beneficial in the context of systemic inflammation, including perhaps, sepsis.

In the present study, IL-10 expression was increased in pretreated animals for the first three hours following LPS exposure and decreased thereafter. In association with the higher levels of IL-10, the levels of both TNF-α and IL-6 were substantially lower in animals pretreated with amylin compared to those that received only LPS. The physiologic significance of these findings was evident in the attenuated levels of illness severity and lung injury in the pretreated animals across the entire study period. Thus, amylin pretreatment may act by increasing the degree and duration of IL-10 expression which might in turn, attenuate TNF-α and IL-6 expression, thereby mitigating systemic inflammation and end-organ damage.

The overall notion that amyloidogenic hexapeptides might possess anti-inflammatory properties has been tested previously, albeit not in the context of systemic inflammation. First, the self-associating regions within these molecules have been shown to serve a chaperone function for denatured proteins [22, 23, 40]. Second, by showing that these peptides bound approximately 70 pro-inflammatory proteins (>50% members of acute phase, coagulation, and complement pathways) in the sera of patients with multiple sclerosis, rheumatoid arthritis, and amyloidosis and in mice with EAE, an inflammation modulatory role was demonstrated for amyloidogenic fibrils [24]. Finally, two distinct pathways, including expression of type 1 IFN by peripheral dendritic cells and the reduced expression of TNF-α, IFN-γ, and IL-6 were shown to contribute immunosuppression by amyloidogenic fibrils [21].

Taken together, studies of amyloidogenic hexapeptides have given rise to a contrarian concept that amyloid-forming peptides and proteins may serve an anti-inflammatory role [1, 10–17]. Our lab has previously corroborated this theory in experimental autoimmune encephalomyelitis [6, 21, 22, 25]. In this study, we broadened the focus of immune-suppression by amyloid-forming peptides to systemic inflammation. The rationale for the current experimental design derives from the observations of Kurnellas *et al*. wherein administration of amyloidogenic fibril (i.p.) triggered an exodus of B-1a lymphocytes to secondary lymphoid organs where the primary anti-inflammatory cytokine, IL-10, was produced, a process that evolved over a 4-hour time frame [25]. Based on this finding, we established an experimental model wherein animals were pre-treated with hexapeptide amylin_28-33_ at least 6 hours prior to LPS (i.p.) administration.

The observation that animals treated with the hexapeptide were less severely ill superimposed upon well-preserved lung barrier function, favorable histology and no increase in apoptosis argues compellingly that the strategy possesses physiologically relevant implications. IL-10 modulates pulmonary inflammation that occurs in the context of pneumococcal infection by constraining the expression of TNF-α, IFN-γ, and IL-6 [41]. Disruption of endothelial barrier function in the lung, and more generally throughout the vasculature, is a critical determinant of morbidity and mortality in the context of sepsis [42]. Preserved barrier function and decreased apoptosis in the pretreated animals provides direct evidence that the hexapeptide amylin_28-33_ can protect the endothelium from disruption in the presence of systemic inflammation.

Relative to a potential therapeutic application, the present strategy of pretreatment with amyloidogenic hexapeptides is limited owing to the unpredictable nature of sepsis. However, there are populations at high risk of inflammation wherein an anti-inflammatory molecule that does not mitigate anti-infective properties, might be highly beneficial. Clinical scenarios wherein inflammation occurs within a predictable timeframe include cardiopulmonary bypass, stem cell transplantation or abdominal surgery. The present data provide support for evaluating amylin hexapeptide in the context of alterative models of inflammation. Whether other amyloidogenic hexapeptides, such as tau, HspB5, PrP possess anti-inflammatory properties remains to be determined.

Thus, we conclude that amylin_28-33_ possesses previously undescribed anti-inflammatory properties. The hexapeptide molecule, amylin_28-33_, can modify systemic inflammation and mitigate end-organ damage as demonstrated by decreased illness severity and mortality. Moreover, pro-inflammatory cytokines are decreased while anti-inflammatory molecules are increased providing some hint at the mechanism whereby pretreatment with amylin_28-33_ protects endothelial barrier function in the lung and presumably diminished end-organ injury. Further study is needed to determine the mechanism that underlies the immune modulation. We speculate that amylin_28-33_ might be a novel therapeutic tool to attenuate disease severity in patients at high risk for inflammation.

## Supporting information

S1 Video Pretreatment with amylin reduces illness severity in an LPS-induced systemic inflammatory mouse model. Adult female B6 mice were pretreated with 20 μg amylin at 12 h and 6 h prior to i.p. administration of LPS; control mice received no pretreatment. All mice were subsequently inoculated with LPS (i.p.) at a dose of 20 mg/kg at time 0. Mice were assessed for illness severity and mortality as described previously. Presented here are representative recordings of illness severity at 48 hours post-LPS administration (i.p.) in (A) control mice and (B) amylin pretreated mice.
(ZIP)

S1 Data Raw data files for amylin study. Enclosed are the raw data used to generate Figs 1 to 5 using Prism 7 for Mac OS X (version 7.0b) software.
(ZIP)
